# The effect of retaining intact posterior capsule in congenital cataract surgery in children aged 4–8 years

**DOI:** 10.1186/s12886-021-02098-9

**Published:** 2021-09-11

**Authors:** Jing Shang Zhang, Jin Da Wang, Mayinuer Yusufu, Kai Cao, Shan Shan Jin, Ying Xiong, Jing Li, Xiu Li Sun, Shu Ying Chen, Zhen Yu Liu, Jing Fu, Li Li, Qi Sheng You, Xiu Hua Wan

**Affiliations:** 1 Beijing Institute of Ophthalmology, Beijing Tongren Eye Center, Beijing Tongren Hospital of Capital Medical University, Beijing Key Laboratory of Ophthalmology & Visual Sciences, Beijing, 100005 China; 2 Beijing Tongren Eye Center, Beijing Key Laboratory of Ophthalmology and Visual Sciences, Beijing Tongren Hospital of Capital Medical University, Beijing, 100005 China; 3 National Key Discipline of Pediatrics, Ministry of Education, Department of Ophthalmology, Beijing Children’s Hospital, Capital Medical University, Beijing, 100045 China; 4 Casey Eye Institute, Oregon Health Science University, Oregon, USA

**Keywords:** Children, Congenital cataract, PCO, Intact posterior capsule

## Abstract

**Background:**

The present study sought to observe the effect of retaining intact posterior capsule in congenital cataract surgery in children aged 4–8 years.

**Methods:**

This is a retrospective case control study. Seventy-seven children (130 eyes) aged from 4 to 8 years who underwent cataract surgery were divided into two groups. In Group A, 50 eyes underwent phacoemulsification, intraocular lens implantation and posterior capsule capsulotomy combined with anterior vitrectomy. In Group B, 80 eyes underwent cataract phacoemulsification and intraocular lens implantation. The postoperative visual acuity and the rate of complications were compared.

**Results:**

In all patients, cataract surgeries were performed evenly without intraoperative complications. The follow-up time ranged from 6 months to 42 months. No apparent visual axis opacity was detected in group A during the follow-up. By the last visit, apparent visual axis opacity was detected in 31 eyes (38.75%) in group B. Among them, 9 eyes (29.03%) with mild posterior capsule opacification (PCO) were treated with Nd:YAG laser, 3 eyes (9.68%) with thick proliferative membranes were treated with posterior capsule capsulotomy combined with anterior vitrectomy and proliferative membranes in 19 eyes (61.29%) were completely aspired and the posterior capsule was retained. During follow-up, only 2 (6.45%) eyes had PCO recurrence and were treated with Nd:YAG laser. The visual acuity was significantly higher than that before surgery in all patients.

**Conclusions:**

For older children, the incidence of PCO will be low even if intact posterior capsule is retained. Either Nd:YAG laser or surgical treatment for PCO will be able to maintain good vision.

## Background

Congenital cataract is one of the main causes of the avoidable blindness in children [1, 2]. At present, the main treatment option for congenital cataract is phacoemulsification with or without intraocular lens (IOL) implantation or posterior capsulotomy in the light of the age. With the development of surgical techniques, surgical equipment and IOL, success rate of pediatric cataract surgery has been increasing. However, there are still many postoperative issues to be dealt with [3], such as postoperative inflammatory reaction, prolongation of eye axis after cataract extraction, the calculations of implanted intraocular lens, secondary glaucoma, posterior capsule opacification (PCO), and amblyopia correction.

The incidence of PCO in adults is 7.7–41.0%, while almost 100% in children. It impairs the transparency of visual axis and subsequently results in amblyopia and strabismus, which may seriously impair the establishment of normal visual function in children. Currently, posterior continuous curvilinear capsulorhexis with anterior vitrectomy are commonly performed to prevent PCO during pediatric cataract surgery [4, 5], while the indication and surgical procedure were still controversial. Though the posterior continuous curvilinear capsulorhexis with anterior vitrectomy can effectively reduce the incidence of PCO, it may increase the postoperative inflammation in children and also the formation of proliferative membrane [6–8]. In addition, the manipulation of the vitreous body may lead to postoperative macular edema and retinal detachment [4, 9]. Although the posterior continuous curvilinear capsulorhexis alone and the optic capture technique (the IOL haptics placed in the capsular fornix after continuous curvilinear capsulorhexis) could reduce the incidence of PCO to a certain extent, there is indeed a significant learning curve with regard to the mastery of the optic capture technique. Furthermore, because the 3-piece foldable IOLs are most suitable for this technique, choosing this technique might preclude the use of certain IOLs such as toric IOLs, which at present are mainly available in the 1-piece design [10]. Therefore, several alternative approaches have been introduced to manage posterior capsule in cataract surgery [3, 11]. Nevertheless, the proper surgical procedure for children at different ages is still controversial.

For children aged 4–8, given the better development and cooperation, the incidence of PCO after retaining the posterior capsule is relatively lower compared with infants [4, 12–14]. For children with fast refractive changes, the retained posterior capsule is a guarantee for IOL exchange [15]. However, whether the effect of reoperation and laser treatment after surgery with an intact posterior capsule is better is yet to be verified. Therefore, it is very important to observe the occurrence of PCO, the safety and the effect of the PCO treatment for children aged 4–8 with intact posterior capsule. It is important to keep the intact posterior capsule during the operation.

In this study, patients were divided into two groups by undergoing even cataract phacoemulsification and intraocular lens implantation with or without posterior continuous curvilinear capsulorhexis and anterior vitrectomy. The Nd:YAG laser and surgery were further performed on children with PCO. The effect of retaining intact posterior capsule on the treatment of congenital cataract in children 4–8 years old was analyzed.

## Methods

### Participants

This was a retrospective case control study. The study was approved by the Ethics Board of the Beijing Tongren Hospital. The study adhered to the tenets of the Declaration of Helsinki. Patients or the public were not involved in the design, or conduct, or reporting, or dissemination plans of our research. All informed consents were obtained from the patients’ guardians before surgery, including the use of their medical data in research.

**Fig. 1:**
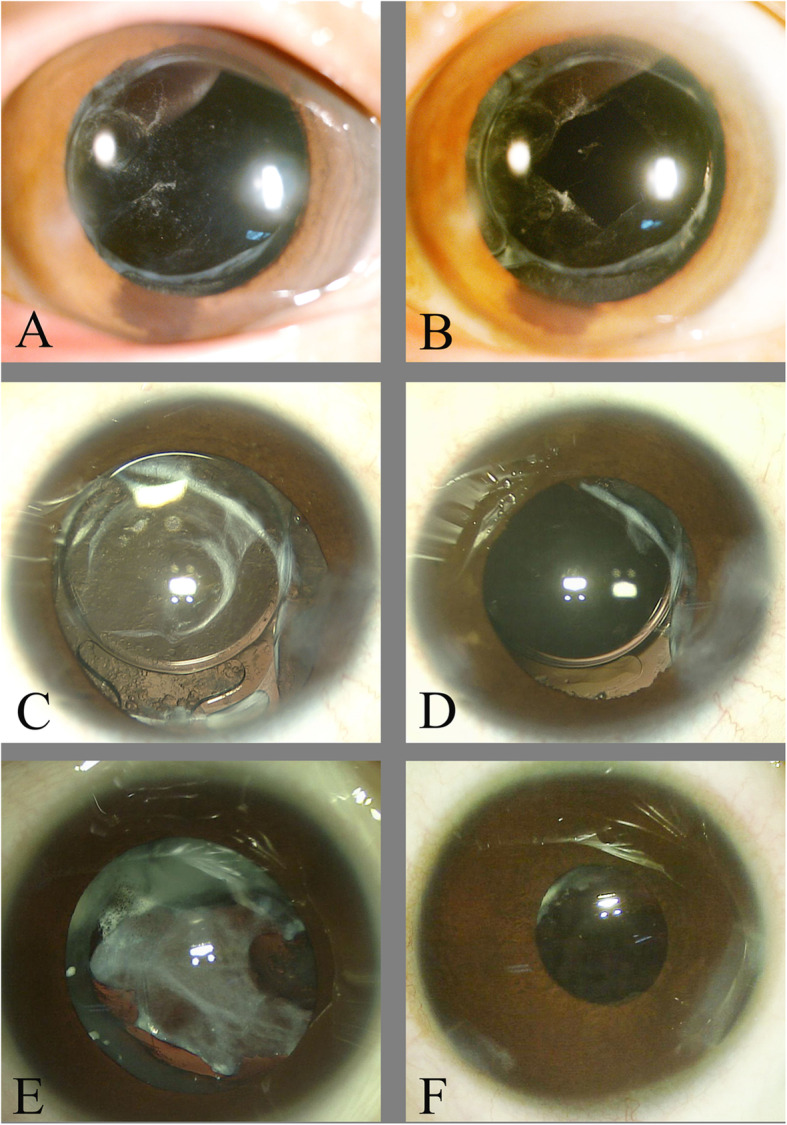
The condition of posterior capsule of different degrees of PCO treated with different methods. **A** Mild PCO; **B** After Nd:YAG laser; **C** Moderate PCO; **D** After posterior capsular proliferative membrane aspiration combined with posterior capsular polishing; **E** Severe PCO; **F** After posterior capsule capsulotomy combined with anterior vitrectomy

Inclusion criteria: Children aged 4–8 received congenital cataract surgery combined with IOL implantation and had been followed up for over 6 months. Exclusion criteria: Patients with fundus diseases, glaucoma, keratopathy, and systemic diseases were excluded. 77 children (130 eyes) who underwent cataract surgery from January 2015 to June 2019 were collected, including 37 boys (61 eyes) and 40 girls (69 eyes). At the time of surgery, the age ranged from 4 to 8 (means ± SD 5.88 ± 1.40) years. Before the surgery, the uncorrected visual acuity was logMAR 0.30–2 (means ± SD 1.12 ± 0.56) and the best corrected visual acuity was logMAR 0.22–2 (means ± SD 0.88 ± 0.25). The patients were divided into group A and group B. In Group A, 50 eyes underwent phacoemulsification, intraocular lens implantation and posterior capsule capsulotomy combined with anterior vitrectomy were included. In Group B, 80 eyes underwent cataract phacoemulsification and intraocular lens implantation without posterior capsule capsulotomy and anterior vitrectomy were included. During follow-up posterior capsular proliferative membrane aspiration combined with posterior capsular polishin, Nd:YAG laser, or posterior capsule capsulotomy combined with anterior vitrectomy was performed for children who developed PCO based on the severity of PCO. The severity of the PCO was graded as: mild PCO with thin proliferative membrane, moderate PCO with pearl-like proliferation, and severe PCO with thick proliferative membrane, posterior capsular fibrosis, and proliferation of cortical substance.Nd:YAG laser was performed for children with mild PCO, the posterior capsular proliferative membrane aspiration combined with posterior capsular polishing was performed for moderate PCO, and posterior capsule capsulotomy combined with anterior vitrectomy was performed for severe PCO (Fig. 1).

Preoperatively, all patients underwent comprehensive examinations including slitlamp biomicroscopy, refraction, intraocular pressure, corneal endothelium count, IOL-Master, B-scan ultrasonography, fundus photography and optic coherence tomography (OCT). Moreover, SRK-T formula was used to calculate the diopter of IOL. A foldable one-piece HOYA was implanted in the capsular bag and the IOLs power were 22–27.5D (means ± SD 24.60 ± 1.31D). The patients were regularly followed after surgery.

### Surgical methods

All the operations were performed under general anesthesia by the same doctor (XHW). Prior to operation, tropicamide eye drops were administered for full mydriasis and oxybuprocaine for topical anesthesia. Subconjunctival injection of dexamethasone was performed after surgery, and tobramycin dexamethasone eye drops were used for 2 weeks.

### Cataract phacoemulsification combined with IOL implantation

In brief, a conventional clear corneal incision 2.2 mm in diameter was created and the anterior capsular continuous curvilinear capsulorhexis was made 5–6 mm in diameter. After phacoemulsification of cataract (Infiniti ozil, Alcon), the intraocular lens was implanted into the capsular bag. After viscoelastic material was aspirated, the anterior chamber was reformed with balanced solution hydration at the incision border.

### Cataract phacoemulsification, intraocular lens implantation and posterior capsule capsulotomy combined with anterior vitrectomy [16]

Besides the abovementioned procedures, the phacoemulsification system was changed to the vitrectomy mode. The infusion needle was inserted into the side incision, the 23-gauge anterior vitrector handpiece was placed into the main incision, and the infusion needle and the anterior vitrector handpiece were all placed behind the IOL. The posterior capsulotomy and anterior vitrectomy were performed by the anterior vitrector handpiece.

### Posterior capsular proliferative membrane aspiration combined with posterior capsular polishing

In brief, the posterior capsule pearl-like turbidity or thinner proliferative membrane was sucked, and the posterior capsular membrane was polished by I/A.

### Nd:YAG laser capsulectomy [4]

In brief, the pupil was dilated by the compound tropicamide eye drops. A slit lamp microscope was used to observe the gap between the intraocular lens and the posterior capsular cavity before surgery. The laser was performed by the Opto Yag laser (OPTOTEK Medical). The posterior capsule ring was cut with a diameter of about 4–5 mm. Postoperative local routine non-steroidal anti-inflammatory eye drops were used for 2 weeks.

### Posterior capsule capsulotomy combined with anterior vitrectomy [4]

In brief, the space between the posterior capsule and the IOL was filled with a viscoelastic. Then the infusion needle was inserted into the 15-degree side incision, the 23G anterior vitrector handpiece was placed into the main incision, and the infusion needle and the vitrector handpiece were all placed behind the IOL. The posterior capsulotomy and anterior vitrectomy were performed by the anterior vitrector handpiece. The posterior capsule ring was cut with a diameter of 4–5 mm.

#### Statistical analysis

Statistical analysis was performed using Statistical Package for the Social Sciences (SPSS, version 20.0). Paired Student’s t-test was used to compare visual acuity before and after surgery. *P* < 0.05 was considered statistically significant.

## Results

### Postoperative review and treatment

In all patients, all cataract surgeries were completed successfully without intraoperative complications. The success rate of intraocular lens implantation in the capsular bag was 100% (130/130). The patients were followed for 6–42 (average 10.87 ± 7.30) months. Patients in group A were younger than those in group B. There was no apparent visual axis opacity in group A until the last visit. There were 31 eyes with apparent visual axis opacity (38.75%, 31/80) detected in group B during the follow-up of 3–36 (average 11.54 ± 8.06) months. Among them, 9 eyes with mild PCO and who could cooperate with Nd:YAG laser were treated with Nd:YAG laser. Surgical treatment was performed in 22 eyes with severe PCO (16 eyes) and in those children who or failed to cooperate with laser (6 eyes).Among them, 3 eyes with thick proliferative membranes were treated with posterior capsule capsulotomy combined with anterior vitrectomy. In other 19 eyes proliferative membrane was completely aspirated from the posterior capsule an the posterior capsule was retained.At the last visit, only 2 eyes had PCO reoccurrence and were treated with Nd:YAG laser. No patient had obvious PCO at the last follow-up visit.

### Postoperative visual acuity

All best corrected visual acuity improved at 1 month postoperatively compared with those before surgery (Tables 1 and 2). Uncorrected visual acuity and corrected visual acuity at the last follow-up were significantly higher than those preoperatively. The improvement of visual acuity in the last follow-up after operation is higher in group A than in group B (see Table 3), which indicates that the visual acuity is better maintained after posterior capsule resection.

**Table 1:** The comparison of preoperation and postoperation for the eyes underwent phacoemulsification intraocular lens implantation and posterior capsule capsulotomy combined with anterior vitrectomy. (*n* = 50)

	Preoperation	Postoperation	t	*P*
Uncorrected visual acuity (logMAR)	1.43 ± 0.50	0.50 ± 0.17	12.44	< 0.001
Corrected visual acuity (logMAR)	0.96 ± 0.11	0.29 ± 0.15	25.81	< 0.001
Intraocular pressure (mmHg)	15.94 ± 3.11	15.52 ± 1.95	0.81	0.421
Corneal endothelium number(n/mm^2^)	3335.70 ± 362.04	2896.40 ± 339.99	6.26	< 0.001

**Table 2:** The comparison of preoperation and postoperation for the eyes underwent cataract phacoemulsification and intraocular lens implantation. (*n* = 80)

	Preoperation	Postoperation	t	*P*
Uncorrected visual acuity (logMAR)	0.92 ± 0.50	0.48 ± 0.27	7.04	< 0.001
Corrected visual acuity (logMAR)	0.71 ± 0.26	0.34 ± 0.31	7.983	< 0.001
Intraocular pressure (mmHg)	15.65 ± 4.03	15.71 ± 4.83	−0.089	0.929
Corneal endothelium number(n/mm^2^)	3387.36 ± 414.72	2928.11 ± 463.84	6.60	< 0.001

**Table 3:** The comparisons between group A and group B

	Group A	Group B	t	*P*
Cases(eye)	50	80		
Follow up time(month)	9.80 ± 5.82	11.54 ± 8.06	−1.32	0.188
Age at operation(year)	4.82 ± 1.08	6.54 ± 1.16	−8.44	< 0.001
^a^Uncorrected visual acuity (logMAR)	−0.93 ± 0.48	−0.44 ± 0.43	−6.01	< 0.001
^a^Corrected visual acuity (logMAR)	−0.67 ± 0.16	−0.36 ± 0.31	−6.45	< 0.001
^a^Intraocular pressure (mmHg)	−0.42 ± 3.63	0.06 ± 4.83	−0.61	0.545
^a^Corneal endothelium number(n/mm^2^)	− 439.30 ± 177.17	− 459.25 ± 464.44	0.36	0.719

^a^ The results are equal to the last follow-up value minus the preoperative value

### Complications

No visual axis opacity and other complications occurred in group A. There was no recurrence of PCO after Nd:YAG laser treatment and posterior capsule capsulotomy combined with anterior vitrectomy in group B. Two eyes recurred PCO after the posterior proliferative membrane aspiration, the recurrence rate was 10.53% (2/19). After the treatment with Nd:YAG laser, there was no recurrence of PCO. 1 eye developed secondary glaucoma, and the IOP returned to normal after treatment with hypotensive eye drops. There was no significant optic nerve damage. No patient showed postoperative complications such as endophthalmitis, corneal endothelial decompensation, macular edema, or retinal detachment.

## Discussion

The maintenance of visual axis transparency after cataract surgery in children is very important for development of normal visual function [3]. But for children after cataract surgery, especially under 4 years old, the intact posterior capsule may lead to 100% occurrence of PCO [4], which seriously affects the recovery of postoperative visual function. Therefore, post-capsular capsulotomy combined with anterior vitrectomy has becoming the first choice. However, the incidence of PCO is relatively low in children over 4 years old, and retaining a complete posterior capsule can reduce intraoperative time, surgical trauma and postoperative inflammatory response. Moreover, the absence of anterior vitrectomy can reduce the interference of the vitreous, which may cause retinal detachment. It is worth noting that, PCO can be treated with Nd:YAG laser for children above 4 years old. If Nd:YAG laser are not available, secondary proliferative membrane removal surgery can be performed. Children will obtain a good visual acuity. In addition, the recurrence rate of PCO is also relatively low [17].

The cataract extraction and posterior capsule capsulotomy combined with anterior vitrectomy were proposed for children with cataracts to prevent the occurrence of PCO and avoid the need for Nd:YAG laser treatment. This method was first proposed by Parks [18] in 1983, and some subsequent studies [19, 20] also suggested this method could effectively prevent the occurrence of PCO. The utmost benefit is that the PCO can be prevented, because there is high PCO incidence with the intact posterior capsular membrane remains [21–23]. Then, the combination of surgeries can reduce the operations times, as well as the economic burden of patients. With the development of minimally invasive vitreous surgery techniques, some researchers proposed that using a minimally invasive posterior vitreous surgery system to treat PCO in children could achieve better surgical results [24]. Some researchers believe that entering the vitreous through the pars plana, together with a 25G vitrector handpiece will reduce the surgery trauma, and the postoperative children recover well and the inflammation is slight [25, 26].

However, there are still controversies on the indications and surgical procedures of posterior capsule capsulotomy combined with anterior vitrectomy [4]. Some researchers believe that there may be certain risks associated with combined surgery [9, 27, 28]. Combined surgery requires good surgical skills and a long operation time. After the removal of the posterior capsule and the anterior vitreous, the vitreous is linked directly with the anterior chamber, which promotes the release of inflammatory factors from the retinal pigment epithelium to the vitreous cavity, and increases the risk of surgical infection and postoperative inflammation. At the same time, the manipulation of the vitreous may cause increased postoperative macular edema and retinal detachment [4, 9]. However, in this study, none of the children who underwent anterior vitrectomy developed cystoid macular edema or retinal detachment.Therefore, although the occurrence of these complications is possible, the incidence is indeed very low.

For older children, if the posterior capsule were kept during the first surgery, the incidence of PCO will be relatively lower than that of younger children. The incidence of PCO in this study was 38.75%, while the incidence of PCO in other studies is 36.4–100% [4, 29]. In addition, Nd:YAG laser can be performed to treat PCO and reduce the impact to the vitreous body. Children can also obtain better vision and there is no need of general anesthesia. In this study, the secondary surgery rate was 27.50%(22/80) in children with intact posterior capsule, which is lower than that of young children (42.86%, 27/63) [17], and they ultimately maintain good postoperative visual acuity.

The incidence of PCO in children is high. Almost all children will develop PCO after surgery and the onset is early. Some cases can develop in the first week after surgery. At the same time, as children are not able to complain about the discomfort, the treatment is often delayed [14, 30, 31]. Once PCO is formed, it should be treated early to promote visual function recovery, avoid amblyopia, and maintain stereoscopic vision [29]. In this study, for patients with mild PCO, Nd:YAG laser treatment was performed early. For severe PCO, posterior proliferative membrane aspiration combined with posterior capsule polishing or posterior capsule capsulotomy combined with anterior vitrectomy should be applied according to the severity of PCO and the age of the child.

The treatments for PCO may lead to different clinical outcomes [32]. Nd:YAG laser treatment of PCO is a common treatment method. The advantage is that it will cause less damage, less inflammatory reaction, and less vitreous interference. It can be used for older children. However, Nd:YAG laser therapy also has its drawbacks. First, it may also transient intraocular pressure (IOP) elevation [33] and cystoid macular edema [34]. In addition, the access to instruments is limited and the compliance in young children is not ideal. Moreover, the Nd:YAG laser therapy has a high incidence of IOL damage in young children. What’s more, there is a chance that, without the posterior capsule, residual lens fibers would lead to the development of secondary opacity membranes by migrating to the intact vitreous surface [4]. A study by Hutcheson et al. reported that 57% patients who received Nd:YAG laser capsulotomy developed visual axis obscuring opacity again, and 17% patients needed to undergo a third laser therapy [35]. Based on the above characteristics, it is very important to choose the timing of the treatment for PCO. Therefore, once the intraocular inflammation after cataract surgery is controlled, Nd:YAG laser should be performed as soon as possible, as the posterior capsule is thin, brittle, and easy to be cut by the laser at the initial phase. Once the capsular fibrosis and thickening to a certain extent, a higher energy is inevitable, which may increase the difficulty of treatment and of the post-operative complications [36]. In this study, during the follow-up after the operation, all children were treated with Nd:YAG laser promptly as long as they were eligible, once it was found that the PCO affected their vision. For the Nd:YAG laser treatment, the right energy should be chosen to minimize the damage of the intraocular lens, and the size of the incision hole should be of the appropriate size. For children with thicker capsules, more energy would be required, thus the lens may be damaged. However, it did not significantly affect the children’s vision after treatment. It is recommended as soon as possible after the occurrence of PCO, in order to reduce the damage and improve the patient’s vision, but when children do not cooperate well or the proliferation is severe, surgery is required [11].

For PCO treatment, methods should be selected based on different conditions [17]. In this study, for order children with severe PCO that cannot be treated with Nd:YAG laser, and the proliferative membrane is not rigid, proliferative membrane aspiration and posterior capsule polishing is recommended, which can reduce the anterior vitreous interference and reduce the inflammatory response. After the operation, the visual acuity of the children can be significantly improved. In addition, the postoperative inflammatory reaction is lighter than that after the anterior vitrectomy. However, the recurrence rate of the PCO is relatively high. If necessary, a further Nd:YAG laser treatment is needed. As Nd:YAG laser treatment is not available in later-stage patients with severe PCO and younger children, or in patients with nystagmus, posterior capsule capsulotomy combined with anterior vitrectomy in the optic axis area may be an alternative. Several reports have confirmed that the intact anterior membrane of the vitreous body provides a scaffold for cell proliferation, and can still form a fibrous membrane causing opacity of the visual axis without the posterior capsule [4]. The posterior capsular capsulotomy combined with anterior vitrectomy was performed in the present study, which provide a certain gap between the vitreous and IOL and is difficult to form PCO. Thus, the method can effectively reduce the recurrence rate of PCO.

The current study has some limitations. Firstly, the sample size of the study is relatively small, and a multi-center study with a large sample should be carried out in the future. In addition, and the age groups with small age intervals should be further studied to determine the most appropriate surgical treatment strategy for each specific age. Second, this research mainly focused on the visual acuity after surgery and the occurrence of related complications such as PCO and glaucoma. The occurrence and treatment of postoperative strabismus and amblyopia were not further discussed in this article, which will be explored in our future research.

In this study, the cataract surgery for children aged 4–8 with intact posterior capsule could retain the complete structure of posterior capsule and reduce the probability of complications of anterior vitrectomy. Although the incidence of PCO was increased for the younger children with intact posterior capsule, the Nd:YAG laser could be given in time after the occurrence of PCO for the cooperative children by the follow-up, and the same good effect could be achieved. Even if serious PCO could not be treated by Nd:YAG laser, posterior capsular proliferative membrane aspiration combined with posterior capsular polishing can be given which could retain good conditions for IOL replacement due to refraction change in later period, or the posterior capsule capsulotomy combined with anterior vitrectomy can be performed in rare cases, finally the children also could obtain the good effect. However, for the older children the incidence of PCO is relatively lower, and the Nd:YAG laser can be basically performed to get the good vision effect and high safety. But the choice of operation methods and the division of age need to be defined accurately by further longer large sample random clinical observation and in-depth discussion.

## Conclusions

Phacoemulsification combined with posterior capsular capsulotomy and anterior vitrectomy for congenital cataract can effectively prevent the occurrence of PCO. For older children, the occurrence rate of PCO will be lower if posterior capsule is kept after cataract surgery. It will also reduce potential risk of complications. Either Nd:YAG laser or surgical treatment will be able to maintain good vision for after PCO. Age is an important reference for selecting surgery type in pediatric cataract.

## Data Availability

The datasets used or analysed during the current study are available from the corresponding author on reasonable request.

## Abbreviations

PCO: Posterior Capsule Opacification
IOL: Intraocular Lens
OCT: Optic Coherence Tomography
